# Massive corneal-epibulbar dermoid managed with pre-descemetic DALK and SLET

**DOI:** 10.3205/oc000178

**Published:** 2021-02-05

**Authors:** Dharamveer Singh Choudhary, Nikhil Agrawal, Maya Hada, Nidhi Paharia

**Affiliations:** 1 Department of Ophthalmology, SMS Medical College, Jaipur, India; 2 Department of Ophthalmology, AIIMS, Bathinda, Punjab. India

## Abstract

Management of large corneal-epibulbar dermoids always poses a challenge to the surgeons due to deeper corneal involvement. Also, there is a risk of limbal stem cell deficiency and formation of pseudopterygium with larger areas of limbal involvement. We report a novel surgical technique for the management of giant corneal-epibulbar dermoid with pre-descemetic deep anterior lamellar keratoplasty (DALK) and simple limbal epithelial transfer (SLET).

## Introduction

Limbal dermoids are the most commonly occurring choriostoma in the epibulbar region [1], [2]. It may or may not be associated with preauricular tags, palpebral coloboma, Goldenhar syndrome (preauricular fistulae, preauricular appendages, and epibulbar dermoids or lipoder-moids), and the mandibulofacial dysostosis of Franceschetti syndrome [3].

The treatment is particularly challenging in young children because of several concerns, such as a tendency for a low visual acuity and astigmatism-related amblyopia. In the past, different surgical techniques for the removal of dermoids have been described. These techniques include bare excision, amniotic membrane transplantation, lamellar and even penetrating keratoplasty. However, the management depends on the tumor size, the tumor growth rate, and the involved areas.

We present a surgical technique for a giant limbal dermoid, managed by dermoid excision with manual deep anterior lamellar keratoplasty (DALK) with simple limbal epithelial cell transfer.

## Case description

**Figure 1:**
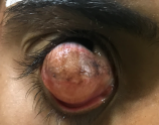
Giant limbal dermoid covering the cornea with pigmentation and fine hairs

Herein, we describe the management of a giant dermoid in a 24-year-old female (Figure 1 (Fig. 1)). The dermoid had the dimensions of 17 mm x 14 mm, straddling horizontally from the lateral canthus to the medial canthus and vertically from the superior one third of the cornea to the inferior fornix. It had a keratinized surface with fine hairs. Slit lamp evaluation showed that the upper margin of the dermoid was extending into the anterior stromal layers. No other systemic association was found on examination. Visual acuity OD was light perception present with accurate projection of rays, and in OS it was 20/20. The patient was planned for right-eye limbal dermoid excision with anterior lamellar keratoplasty (ALK) with limbal reconstruction by simple limbal epithelial transplant (SLET).

### Surgical technique

**Figure 2:**
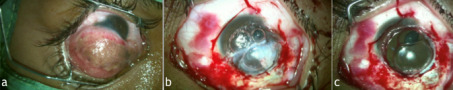
Intra-operative pictures showing (a) limbal dermoid sparing superior one-third of the cornea; (b) deeper involvement of corneal layers visualized after dermoid excision; (c) Descemet’s membrane was exposed after manually dissecting deeper stromal layers.

**Figure 3:**
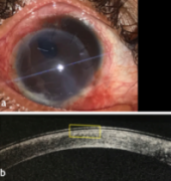
Postoperative pictures; (a) graft sutured to host with 10-0 nylon; (b) OCT shows minimal interface haze.

Surgery was conducted under local anaesthesia, and dermoid excision (Figure 2a (Fig. 2)) was initiated. Limited conjunctival peritomy was done superior and temporally, and blunt dissection of the lesion was carried out. Lamellar dissection of the lesion from the cornea was done with a crescent blade. However, deep extension of the dermoid in the posterior stromal layers was noted (Figure 2b (Fig. 2)), which lead to a change of plan from ALK/amniotic membrane grafting to manual pre-descemetic DALK. Manual dissection was carried out to expose the Descemet’s membrane (Figure 2c (Fig. 2)). After this, a donor tissue 9.5 mm in size was prepared by scraping the endothelium and trephine. Thereafter, the tissue was secured with the host with a 10-0 nylon suture (Figure 3a (Fig. 3)).

Limbal stem cells were harvested from the normal eye of the patient. Multiple small pieces of limbus were arranged concentrically around the visual axis with fibrin glue. Thereafter, the contact lens was placed over the graft.

On day 1 postoperatively, the graft was clear, but there was interfacial fluid collection which resolved after 3 weeks (Figure 3a (Fig. 3), Figure 3b (Fig. 3)). At 1 month follow-up, BCVA was finger counting close to face (FCCF), attributed to amblyopia. There was no pseudopterygium formation or any graft-related complication at 6 months follow-up. Despite of dense amblyopia, the results were cosmetically satisfactory for the patient.

## Discussion

Anatomically, limbal dermoids have been classified in three grades [4]. Grade I limbal dermoids are superficial lesions measuring less than 5 mm and are localized to the limbus. Grade II limbal dermoids are larger lesions covering most of the cornea and extending deep into the stroma down to Descemet’s membrane without involving it. Grade III limbal dermoids, the least common of all the presenting dermoids, are large lesions covering the whole cornea and extending through the histological structures between the anterior surface of the eyeball and the pigmented epithelium of the iris. The surgery is indicated for grade II and grade III limbal dermoids, and primary surgical intervention is indicated even in grade I limbal dermoids if progressive corneal surface decompensation, astigmatism, or encroaching into the optical zone is observed.

Surgical approaches for grade II and grade III limbal dermoids include excision, lamellar or penetrating keratoplasty, and amniotic membrane [5], [6], [7]. However, pseudopterygium formation has been reported after dermoid excision due to limbal stem cell deficiency [8]. Therefore, in our patient with giant limbal dermoid, where the primary concern was a cosmetically acceptable eye ball, SLET [9], [10] was performed along with lamellar keratoplasty and dermoid excision.

Our case not only highlights the importance of timely management of limbal dermoid and surgical expertise to carry out DALK in case of deeper layer involvement of the cornea, but also the role of the limbal stem cell transplant to prevent pseudo-pterygium formation.

## Conclusion

The authors recommend individualized management for cases of large corneal-epibulbar dermoid. Significant cosmetic and visual outcome in challenging cases of giant epibulbar dermoid can be achieved with the described surgical technique.

## Notes

### Competing interests

The authors declare that they have no competing interests.

### Informed consent

Prior informed consent has been obtained from the patient for the documentation of the case for research and academic activities.
